# Leucocytes

**Published:** 1868-11-01

**Authors:** M. Lortet


### CHICAGO MEDICAL JOURNAL.

*Vol. XXV.—NOVEMBER* 1, 1868.—*No.* 21.

### LEUCOCYTES.

*Can they be developed spontaneously in Blastemata? Origin of Leucocytes found in the midst of Blastemata primarily amorphous, isolated in permeable receptacles. By* M. LORTET, M.D., S.D.

TRANSLATED EXPRESSLY FOR THIS JOURNAL, BY WALTER HAY, M.D., ASSOCIATE EDITOR.

THERE are presented to us, at this time, two theories to explain the genesis of anatomical elements. The one assumes as its fundamental doctrine, that cell originates directly from cell; the other, on the contrary, admits that certain organized anatomical elements may originate spontaneously in amorphous blastemata, by and out of expense of the latter. If it were once thoroughly demonstrated that an organism as characteristic as the leucocyte, for example, could originate spontaneously in the midst of a liquid placed under certain particular conditions, this would certainly be a very important conquest by the defenders of the theory of generative blastemata. It is this especial point, moreover, already investigated by several physiologists, which constitutes the subject of this paper. In 1867, M. Onimus published, in the *Journal of Anatomy and Physiology* of Professor Robin, a memoir entitled: "*Experiments upon the genesis of Leucocytes.*" The author affirms that in certain blastemata, entirely free from organized elements, enclosed in receptacles composed of organic membranes, and placed in the interior of wounds effected in animals, after a short interval of time, there are produced spontaneously leucocytes, well organized, and in great numbers. M. Onimus introduces, under the skin of rabbits, little sacs of gold-beaters' leaf, filled with the serosity from fresh blisters. Twelve hours afterward, the serosity is found still transparent, although it has lost its primitive yellow color: already several leucocytes and granulations may be observed. At the end of twenty-four hours, the serosity contains many granulations and leucocytes; after thirty-six hours, it is entirely white, and is composed solely of leucocytes and granulations. Moreover, M. Onimus asserts that, in order that the genesis of leucocytes should occur, it is necessary that the fibrine be not coagulated; for, according to him, neither leucocytes nor any other species of anatomical elements are formed in the serosity of a blister whose fibrine has been precipitated by coagulation. We shall perceive, later, how little the facts which we have observed harmonize with this opinion. From these different experiments M. Onimus deduces the spontaneous generation of leucocytes in fibrinous blastemata placed under peculiar conditions of temperature and of endosmosis.

These experiments are too important to pass by unnoticed; but already the investigations of M. Cohnheim (of Berlin) upon inflammation have enabled us to perceive that M. Onimus has been deceived, not only in his facts, but in the explanation which he gives of them. According to M. Cohnheim, in certain inflammations, leucocytes are not always the result of the proliferation of the nuclei of connective tissue. Frequently they are nothing else than those of the blood which have passed through the capillary walls. This phenomenon is easy to establish; if, in a frog poisoned by curare, the mesentery, irritated simply by contact with the air, be examined microscopically, leucocytes are seen to pass slowly out of the vessels which contain them. So, also, in an inflamed tissue the vascular parietes become fitted to permit the passage of these little organisms, which does not occur in a physiological condition. The leucocytes are elongated, drawn out, bent, changed in form at every instant, just as true amæbæ, which they appear to be, and finish by means of these movements, by penetrating into the fabric of the tissues. In order that this phenomenon may be accomplished, it is necessary that these organisms should be living, or, rather, that they should be found in certain conditions of life, of temperature, and of medium, as we shall, in a moment, perceive.

On the other hand, the results of the experiments published by M. Chauveau in his investigations into the mode of penetration of virulent corpuscles into the organism—results demonstrating that leucocytes are introduced by myriads through membranes plunged into a medium loaded with leucocytes, might cast a doubt upon the truth of the theory of M. Onimus, for the capital importance of the fact affirmed by this physiologist, that is to say, the spontaneous generation in certain blastemata of bodies as highly organized as leucocytes, deserved, indeed, the most serious study. Moreover, in spite of the clearness of the differential results published by this author, according to the nature of the blastema introduced into the receptacles, we have considered it necessary to repeat those experiments, placing ourselves for that purpose in conditions free from any objection.

We have especially experimented with blastemata which we could be assured was a non-fibrinous liquid, and primarily entirely free from leucocytes. The animals upon which wounds were established for the purpose of experiment, were invariably horses and asses; the receptacles destined to contain the blastemata were either pouches made of gold-beaters' skin or the swimming bladders of finches or carps. Membranes which are almost purely fibrous, and whose walls contain no nuclei or cellules which could be confounded with leucocytes. The liquids, which we have placed in these organic vesicles, were,

1st. Pure egg-albumen, which contains only some filamentous traces, and a few vitelline cells.

2nd. Cerebro-spinal fluid, freshly taken from the horse. This liquid, examined carefully with the microscope, shows only a few scattered granules. By chemical tests, very little albumen could be detected.

3rd. Solutions of non-azotized substances, such as gum acacia and sugar. These solutions were carefully examined and exhibited nothing which could be mistaken for leucocytes.

4th. Distilled water.

5th. Bladders, filled with atmosphere only, have been introduced into the wounds.

Receptacles filled with these different liquids were placed in the interior of subcutaneous incisions, freshly made, in the flanks of horses or asses, and left, ordinarily, for twenty-four hours undisturbed. The bladders filled with albumen contained, after twelve hours, a very great number of leucocytes. After twenty-four hours the liquid was entirely purulent. The leucocytes were extremely numerous, large and well-preserved.

With the cerebro-spinal fluid there was the same result; complete purulence at the end of twenty-four hours. Many granulations. This liquid appeared to preserve admirably the leucocytes, which maintained, for a long time, all their typical characteristics.

With the solutions of gum acacia and of sugar, complete purulence after twenty-four hours. The leucocytes were likewise well preserved, but agglutinated amongst themselves into large patches.

With the distilled water, the same result. The liquid had become albuminous by endosmosis. The leucocytes were large and swollen, and their nuclei were very perceptible. Granulations were numerous.

Finally, when the receptacles are distended only with atmospheric air, the leucocytes penetrate as well into their interior. It is necessary, however, to be careful that the internal pressure be not too strong, otherwise the phenomena of penetration are effected with difficulty. These receptacles of air are not filled up entirely, but exhibit only a few drops of pus in their interior, and their membranous walls are, in a manner, stuffed with leucocytes. The swimming bladders of fishes are especially favorable for the establishment of this last fact. They are entirely fibrous, and the fibres which compose them are extremely translucid. Indeed, after remaining twelve hours only in a bleeding wound, there may be seen, upon these bladders, large patches, broad, whitish zones of a milky whiteness, truly purulent. With the aid of the microscope there may be recognized, without difficulty, long lines of leucocytes, ranged one against the other, which are effecting a passage for themselves through the fibres of the tissue as if by violence. When the wound is entirely fresh, when it is very bloody, the receptacles contain, with the leucocytes, hæmatine in sometimes insufficiently large quantity to impart to the liquid contents a bright rose color. The hæmatine must originate from the destruction of a certain number of sanguineous globules; but never have we been able to perceive a single red globule penetrate into the interior of the receptacles.

The pressure exercised by the lips of the wound upon the liquid in which the receptacle is bathed, has evidently no influence upon the penetration of leucocytes. It is easy to relieve these receptacles of this compression by enclosing them in tubes of glass with two ends. In spite of this precaution, these little organisms no less freely introduce themselves into the receptacular cavity.

It is evident that pressure exercised upon the pus of the wound does not enter at all in the production of these phenomena of penetration. In order to be convinced of this, take the swimming bladder of a fish; evert it in such a manner that the internal surface becomes the external, fill it with pus and attach it firmly to one of the extremities of a U shaped tube, into the larger branch of which gently pour some mercury. With this apparatus it may be established that even under the pressure of nineteen centimetres of mercury, maintained during twenty-four hours continuously, not a single leucocyte traverses the membranous pouch. Under augmented pressure the pouches are ruptured, but the purulent globules do not pass out. In order that this penetration might take place, it is evidently necessary that the leucocytes be found in certain conditions of temperature and of vitality. Thus, when the pouches are plunged into an old wound in which there is only creamy, old, and probably altered pus, very few leucocytes are found in their interior, although a small number may always be detected. In this case, however, the phenomena of endosmosis are effected equally well, since distilled water, placed in similar conditions, becomes strongly albuminous. And here is a circumstance extremely important to note, that the more recent and bloody the wound, the more rapid is the penetration, and the more numerous the leucocytes in the pouch.

From the preceding experiments the following conclusions may be deduced :

1st. In an amorphous blastema, enclosed in a permeable pouch, and placed under determinate conditions of osmosis and of temperature, then introduced into a purulent or sanguineous medium, there is no spontaneous generation of leucocytes, but these little organisms pass between the fibres of the membranes, in consequence of the facility with which they can change their form.

2nd. Pressure has no influence upon this penetration.

3rd. The character of the liquid contained within the pouches is altogether a matter of indifference.

4th. It is necessary, in order that the leucocytes may penetrate the membranes, that they be endowed with movements, (Sarcodigma), and that they should be placed in determinate conditions of temperature and vitality.

5th. Leucocytes contained in a recent and bloody wound, penetrate much more rapidly, and in much greater number, than those in an old and purulent wound.

M. Ranvier has made a certain number of experiments which come to the aid of the conclusions of M. Lortet. He placed fragments of the pith of the elder tree under the skin of a certain number of animals, and has determined that leucocytes would penetrate into the interior of this pith from the periphery to the centre. M. Ranvier does not believe in the spontaneous organization of blastemata, but it must be noted that the experiments of M. Lortet, and his own, demonstrate only the facility with which leucocytes can penetrate certain tissue; they prove nothing against spontaneous generation in blastemata.

M. Legros has repeated, finally, the experiments of M. Onimus. He used sacks of gold-beaters' leaf, which had been previously tested under water by insufflation, and which appeared perfectly impermeable. He, moreover, made the experiment with dialyzing paper, and in every case obtained the same result as M. Onimus. He, therefore, believes in the spontaneous generation of leucocytes in blastemata, and asserts that the importance of the amæbori movements have been exaggerated. M. Cornil has been able to assure himself that the substances employed in these experiments, such as gold-beaters' leaf, dialyzing paper, etc., are impermeable only during the first hours of their detention in the liquid. At the end of a certain time they soften and become permeable. If they are examined with the microscope, there are invariably found in them openings, more or less extensive. By permitting a sack of gold-beaters' leaf to remain distended in a vessel filled with pus, the latter speedily insinuates itself through the membrane into the interior of the sack. M. Lortet has stated that, in his experiments, he took great precautions in order that the pouches used by him should be perfectly closed. He thinks, therefore, that in those cases especially on which he used the swimming bladders of fishes, the leucocytes insinuated themselves between the fibres of these bladders, in consequence of their amaboid properties, and by means of microscopic investigations of their walls, he has always been able to recognize lines of white globules passing from the external to the internal surface.

M. Hayem observes that, in the experiments undertaken by M. Onimus and Legros, objection ought be made, not only to the receptacles, but also to the liquid considered as blastema. Indeed, M. Onimus asserts that the condition essential to the success of the experiment consists in the non-coagulation of the sėrosity. Now, M. Valpian assures us that the serum collected in the vesicles of blisters prevents constantly, at thė end of about ten minutes, a fibrinous coagulation.

Mereover, the infiltration of the serum does not retain certainly all the white globules. It is well known that these can pass through a paper filter. There is therefore introduced into the gold-beater's leaf, not only a pretended blastema having already furnished a clot, but, in addition, a liquid containing, perhaps, a certain number of white globules. Moreover, M. Hayem believes, likewise, in the easy penetration of the gold-beater's leaf by the white globules; so that, in his opinion, the contents and the containing membrane are equally inappropriate for the demonstration of the fact which has been advanced.

M. Legros asserts that the serum of blisters coagulates only in a certain number of cases. He adds that the presence of leucocytes in the interior of membranes can be interpreted in a manner totally different from that proposed by M. Lortet.

